# The Association of a Single Nucleotide Variant in *COL5A1* to Early Onset Keratoconus and Pectus Excavatum—Convergence of Extracellular Matrix Pathologies

**DOI:** 10.3390/medicina60060974

**Published:** 2024-06-13

**Authors:** Griffin Bryant, Peyton Moore, Mohanakrishnan Sathyamoorthy

**Affiliations:** 1 Sathyamoorthy Laboratory, Department of Medicine, Burnett School of Medicine, Texas Christian University, Fort Worth, TX 76109, USA; 2 Consultants in Cardiovascular Medicine and Science, Fort Worth, TX 76104, USA; 3 Fort Worth Institute for Molecular Medicine and Genomics Research, Fort Worth, TX 76104, USA

**Keywords:** *COL5A1*, keratoconus, pectus excavatum, single nucleotide variants, collagen matrix proteins

## Abstract

Keratoconus is a bilateral ocular condition characterized by irregularities and the thinning of the cornea. Decreased central corneal thickness is a hallmark of the condition, and numerous genes have played a role in altering corneal thickness and the subsequent development of keratoconus. Variants in the structural and regulatory genes of the extracellular matrix have been highly associated with keratoconus, as well as with pectus excavatum, a chest wall deformity commonly seen in connective tissue disorders. This report describes a patient with a c.1720-11T>A intronic variant in the collagen-encoding gene, *COL5A1,* who was diagnosed with early-onset keratoconus and demonstrated a significant pectus excavatum. This report associates a *COL5A1* variant with these seemingly unrelated phenotypic associations, further advancing the literature on the topic.

## 1. Introduction

### 1.1. Keratoconus

Keratoconus (KC) is characterized as a bilateral, asymmetric eye disease that causes irregularities and the thinning of the cornea. This disease state leads to irregular astigmatism and a decline in visual acuity over time. The current estimated prevalence is broad and is estimated to be between 0.2 and 4790 per 100,000 persons. The current incidence rate is between 1 and 30 cases per 100,000 person/year, with higher rates present in 20- to 30-year-olds. Further, some research suggests that the incidence may be higher among certain ethnic groups in the Middle East and Asia [1]. Keratoconus often presents in the early adolescent years with progression during ages 20 to 30, and has well-known risk factors, including a family history of keratoconus, an atopic history, and eye rubbing [2]. Presenting symptoms include progressive visual blurring and distortion, secondary to developed myopia and high astigmatism. Other presenting symptoms may include photophobia, glare, and monocular diplopia [3]. The pathophysiology of keratoconus is intricate, influenced by a combination of genetic and environmental factors. Key risk factors identified in case–control studies include a family history of keratoconus and eye rubbing [4,5]. Additionally, recent research suggests that nocturnal ocular compression, stemming from inadequate sleeping positions, may also play a role [6]. Various mechanisms have been proposed for keratoconus development, such as the dysregulation of collagen synthesis, slippage of collagen fibrils and lamellae, an elevated expression of collagenolytic enzymes and pro-inflammatory cytokines, compromised antioxidant defenses, thyroid dysfunction, and collagen degradation due to UV exposure [4,7].

A distinct feature of keratoconus is the thinning of the cornea, with an average difference of 75 μm in central corneal thickness (CCT) from normal controls. The genomic loci associated with CCT changes have been found to contain collagen-encoding genes, including *COL1A1, COL1A2, COL8A2,* and *COL5A1* [8]. Numerous other variants have been associated with keratoconus development, including polymorphisms in *VSX1, TGFB1,* and *LOX* [2]. These findings have led to advancements in genetic panels for keratoconus risk stratification; however, more data reporting is required to increase the accuracy of the tests. The most recent advancements in risk stratification for KC are centered around the incorporation of artificial intelligence. These artificial intelligence models utilize machine learning to analyze the high-risk factors associated with KC progression [9]. One such study used AI to interpret corneal tomography obtained through Scheimpflug imaging [10].

The current gold standard keratoconus screening tool is corneal topography [11]. Early-stage keratoconus typically requires the addition of corneal pachymetry for proper diagnosis. These tests allow for not only the diagnosis but the analysis of the severity and progression of the disease [12]. Due to the high variability of the time course for the development of keratoconus signs and symptoms, classifying the severity of keratoconus is an ongoing challenge. The majority of the classification systems currently utilized depend on corneal morphology, though there is still no uniformly agreed upon classification system for keratoconus [13]. The Amsler–Krumeich (AK) system is one of the oldest and most widely used classification systems for keratoconus severity. Central keratometry, scarring, central corneal thickening, and spectacle refraction are all incorporated into this system. The patient’s severity is graded on a stage 1–4 scale and treatment options are explored based on the stage [14]. As previously stated, the treatment for keratoconus is dependent on the classification system utilized and the symptoms present at the time of diagnosis. A corneal transplant is reserved for severe cases of keratoconus that are refractory to treatment with scleral lenses, corneal cross-linking, and refractive surgical procedures [1].

### 1.2. Pectus Excavatum

Pectus excavatum is a chest wall deformity that results in the inward growth of the sternum and ribs, resulting in a depression of the chest. Exercise intolerance is the most common presenting symptom for patients with pectus excavatum. This is likely due to diastolic filling issues, secondary to mechanical compression of the right ventricle by the thoracic depression [15]. The incidence of mitral valve prolapse is significantly increased in patients with PE, though the mechanism is not fully understood [16]. The Haller Index (HI) is the widely recognized standard with which the severity of PE is graded. A CT scan is used to determine the ratio of the transverse diameter of the chest and the anteroposterior diameter of the thorax [17].

The surgical correction of pectus excavatum involves restoring the thoracic silhouette via minimally invasive techniques or, in more severe cases, reconstructing the chest wall with prosthetics [18]. Surgical correction is considered for patients with a significant deformity. Indications for surgical correction include: A Haller index of 3.25 or greater, patients experiencing exercise intolerance, shortness of breath, or chest pain, obstructive or restrictive pulmonary dysfunction, cardiac evaluation that displays murmurs, arrhythmias, mitral valve prolapse, or displacement of the heart [19].

This phenotype may be an inconsequential finding, but it is frequently found in connective tissue disorders [20]. Variants in collagen-encoding genes, such as *COL5A1*, are common findings in connective tissue disorders, due to their role in maintaining the integrity of the extracellular matrix (ECM). *COL5A1* variants are historically associated with ECM-related pathologies, such as classic Ehlers–Danlos Syndrome (cEDS). This disease is inherited in an autosomal dominant fashion and exhibits skin hyperextensibility, abnormal scarring, joint hypermobility, easy bruising, mildly blue sclerae, pes planus, and scoliosis [21]. The disruption of ECM components affects the structure and integrity of the ECM, resulting in phenotypes such as keratoconus, pectus excavatum, cEDS, and various other disease states.

## 2. Case Description

A 76 y/o female with a history of bilateral significant keratoconus since early childhood presented to our practice for a comprehensive cardiac evaluation. We learned that she had undergone multiple bilateral corneal transplants, with a failure of the left eye (OS) transplant in 2021 requiring penetrating keratoplasty (PKP) in 2021. Her most recent corneal exam of the right eye (OD) demonstrated a DSEK clear compact graft, attached 360 degrees, with overlying full thickness PKP, and a 3+ band keratopathy of the inferior cornea. The left eye (OS), which underwent a DSEK transplant in 2019, had a failed full thickness corneal graft, and a central stromal opacity with vascularization present at the GHK with temporal PAS. Both eyes have been treated with prednisolone and she recently underwent a repeat penetrating keratoplasty (PKP #2) of OS in March of 2024.

On our initial physical cardiovascular examination, we noted a significant pectus excavatum and found this to be curious in relationship to her keratoconus, as both are known to have a basis in extracellular matrix protein dysregulation. She had no cardiac or thoracic symptoms and lacked any significant cardiovascular family medical history, including coronary artery disease, aortic aneurysms, pectus excavatum, or aortic dissections. Her pectus excavatum was evaluated by a thoracic CT, which demonstrated a qualitative severe pectus with a Haller index of 2.7. A transthoracic echocardiogram (TTE) with aortic imaging was performed, utilizing a leaning forward protocol; this demonstrated no anatomical or functional cardiac consequence of her pectus by echocardiographic criteria. There was no ascending aortic aneurysm or any other anomalies on her echo exam. The patient was offered clinical genetic testing to screen for pathogenic mutations that would influence clinical decision-making by utilizing a panel of 35 genes associated with the development of ECM related diseases.

## 3. Material and Methods

We used a commercial panel of 35 genes associated with ECM disorders, including “ZNF469, SLC2A10, BGN, SMAD3, FOXE3, TGFBR1, MED12, COL3A1, ACTA2, FLNA, FKBP14, SMAD4, FBN1, COL1A2, MYLK, TNXB (not including exons 32–44), FBN2, MFAP5, COL5A2, TGFB3, CBS, PLOD1, LOX, COL1A1, TGFBR2, MYH11, CHST14, SKI, COL5A1, EFEMP2, TGFB2, NOTCH1, MAT2A, PRDM5, and PRKG1”. The identification of these variants is performed by either Sanger or Next Generation sequencing of coding domains, intronic domains, and untranslated regions. NCBI reference sequence NM_000093.5 was used for COL5A1 analysis. Many methods were used for genetic analysis, including gross deletion and gross duplication investigations to determine the gene copy number for exons that were covered, and the UTR of the genes listed. Further, bait-capture was used for thee enrichment of coding exon sequences via “biotinylated oligonucleotide probes and subsequent polymerase chain reaction and sequencing, and utilizing NCBI reference sequences [22]”. Further, additional Sanger sequencing was performed for regions that were missing or regions with insufficient read depth coverage for reliable variant detection [22]. Standard one-to-one clinical genetic counseling was provided to our patient, as it is to other patients with potential ECM related disorders at our office. Genetic analysis was performed by a CLIA certified commercial lab and the results are confidentially communicated to our program.

## 4. Genetic Results

**Figure 1:**
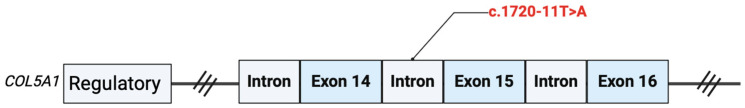
The c.1720-11T>A variant is located in the intron upstream from coding exon 15. This variant has been identified as likely pathogenic due to alterations in the native splice acceptor site and has been associated with cEDS phenotypes in patients [25].

We identified a likely pathogenic variant, c.1720-11T>A, residing in the intron upstream of exon 15 (coding exon) of the *COL5A1* gene (Figure 1). We performed a gnomAD database search and identified the variant as rare based on current population data [23]. This variant results in an intronic T to A substitution of 11 nucleotides upstream from the coding exon 15 in the *COL5A1* gene, and has previously been identified in individuals with cEDS features. An in silico splice site analysis predicts that this variant results in alterations that weaken the native splice acceptor site, leading to defective protein production [24]).

It is important to note that no other genetic alterations were found among the other 35 genes analyzed. Though this is not the first report to associate *COL5A1* mutations with keratoconus or pectus excavatum, this report is the first we are aware of that reports a very likely pathogenic variant in this gene to both clinical phenotypes in the same patient. By doing so, it is also the first to link these seemingly disparate clinical disorders, which we believe will advance the literature associating these genotypic variants to these phenotypes. A further confirmatory, mechanistic study of this variant at the molecular level would be necessary to attribute causation, and we are hopeful our discovery will motivate these investigations.

## 5. COL5A1

**Figure 2:**
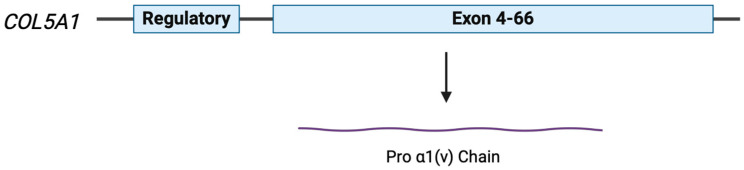
Simplified structure of the COL5A1 gene and protein product. The gene encodes for the pro-α1(v) chain of type V collagen [25].

The *COL5A1* gene encompasses 66 coding exons located on the long arm of chromosome 9 and encodes the alpha-1 chain protein of type V collagen (Figure 2). Alpha-1, alpha-2, and alpha-3 chain proteins make up the trimer of proteins that form the triple-helix structure of type V procollagen. Type V collagen interacts with the structural proteins within the extracellular matrix, providing structural integrity. Type V collagen also combines with Type I collagen, creating a dimeric fibril that is a significant modulator of fibrillogenesis within the skin and cornea [26]. 

Genetic alterations in *COL5A1* in humans are well described in connective tissue EDS. EDS is a family of 11 disorders caused by variations in the *COL* gene. Each manifestation of EDS has symptoms specific to the type of collagen affected, though hyperextensible skin and joint hypermobility are common among nearly all EDS patients. cEDS affects Type V collagen and is caused by a heterogeneous pathogenic variation in *COL5A1* or *COL5A2* [27].

## 6. Discussion

The genetic etiology of keratoconus has not been completely identified, and multifactorial interactions between environmental and genetic factors are suspected. Keratoconus can be associated with genetic diseases, from the mechanical trauma of eye rubbing, mitral valve disorders including prolapse, atopic disorders, the use of contact lenses, and familial diatheses. The identification of the genetic variants responsible for keratoconus development is necessary for improving screening and diagnostic tools. Current research has found an association between keratoconus and these 24 genes: “*COL5A1*, *DOCK9*, *FNDC38*, *COL4A1*, *CRX*, *FOXO1, VSX1*, *COL6A1*, *HGF*, *LOX*, *COL8A2*, *MIR184*, *ZNF469*, *MPDZ*, *COL8A1*, *RAB3GAP1*, *RXRA*, *COL1A1*, *SOD1*, *SPARC*, *CRB1*, *COL4A3*, *PRDM5* and *TGFB1* [28]”. Further exploration of all SNVs reported in these genes correlated with a definitive phenotype will advance the knowledge behind keratoconus and its development. Although reports of SNVs may help to advance screening tools, further mechanistic studies are required to determine the pathogenicity for each.

The genetic etiology of pectus excavatum is undefined, but there is consensus that a genetic basis either underlies or contributes to the phenotypic development. This reasoning stems from the recognition that nearly 40% of patients with pectus excavatum have family members with similar presentations [29]. Of the associated SNVs, many are associated with ECM-related genes. Notably, patients with mutations in the *COL5A1* and *COL5A2* genes have been associated with pectus excavatum development [30]. Disruption in these genes that encode vital components of collagen is thought to result in the loss of integrity of the ECM, resulting in the pectus excavatum phenotype. SNVs in the *COL5A1* gene, although associated with pectus excavatum and keratoconus phenotypes separately, has never been reported in the literature to cause both in a single patient.

The relationship between ECM regulatory and structural genes and proteins, including the *COL* genes and their collagen protein products, and the development of various disease states, continues to be investigated. We hypothesize that disruption in the ECM in patients with dysfunctional collagen products increases the likelihood of both keratoconus and pectus excavatum. This hypothesis is based on the idea that Type V collagen is essential for corneal structure and transparency, as well as for ECM structure and stability in the chest wall. Due to its significant role in maintaining the integrity of the ECM in both the cornea and chest wall, we find it plausible to suggest that the disruption of the normal functioning of the *COL5A1* gene plays a role in disease formation.

The limitations of our study include recognizing that pathogenicity cannot be firmly established without further molecular study; we anticipate that this report will encourage further investigative mechanistic studies. Further, the genetic history of our patient’s family is not available; determining if this *COL5A1* variant was de novo or segregated with disease in the family would further support pathogenicity.

## 7. Conclusions

Keratoconus can lead to significant vision impairment, underscoring the importance of timely diagnosis and treatment. Alongside critical environmental factors such as eye rubbing, an expanding body of research links the disease’s development to novel variants in ECM regulatory and structural genes. Similarly, pectus excavatum has been associated with connective tissue disorders and variants in ECM-related genes, with severe cases requiring surgical intervention. Our patient is the first reported in the literature to present a variant in the *COL5A1* gene associated with both keratoconus and pectus excavatum. This report advances the understanding of variants in ECM regulatory and structural genes with various disease processes, including keratoconus and pectus excavatum, and serves as a potential single cause for these two phenotypes in this patient. Further mechanistic studies exploring *COL5A1* variant pathogenicity will help to improve the current screening tools and diagnostic tools for keratoconus and other ECM related diseases, including pectus excavatum.

## Data Availability

The presented data are available upon request from the corresponding author listed above. Due to privacy considerations, data are not publicly available.
